# Dying in Darkness: Deviations From Data Sharing Ethics in the US Public Health System and the Data Genocide of American Indian and Alaska Native Communities

**DOI:** 10.2196/70983

**Published:** 2025-03-26

**Authors:** Cason D Schmit, Meghan Curry O’Connell, Sarah Shewbrooks, Charles Abourezk, Fallon J Cochlin, Megan Doerr, Hye-Chung Kum

**Affiliations:** 1 Department of Health Policy and Management School of Public Health Texas A&M University College Station, TX United States; 2 Population Informatics Lab School of Public Health Texas A&M University College Station, TX United States; 3 Program in Health Law and Policy School of Public Health Texas A&M University College Station, TX United States; 4 Institute for Healthcare Access Texas A&M University Ft. Worth, TX United States; 5 Great Plains Tribal Leaders Health Board Rapid City, SD United States; 6 Rosebud Sioux Tribe Supreme Court Rosebud, SD United States; 7 Lower Brule Sioux Tribe Lower Brule, SD United States; 8 Sage Bionetworks Seattle, WA United States

**Keywords:** ethics, information dissemination, indigenous peoples, public health surveillance, privacy, data sharing, deidentification, data anonymization, public health ethics, data governance

## Abstract

Tribal governments and Tribal Epidemiology Centers face persistent challenges in obtaining the public health data that are essential to accomplishing their legal and ethical duties to promote health in American Indian and Alaska Native communities. We assessed the ethical implications of current impediments to data sharing among federal, state, and Tribal public health partners. Public health ethics obligates public health data sharing and opposes data collection without dissemination to affected communities. Privacy practices, like deidentification and data suppression, often obstruct data access, disproportionately affect American Indian and Alaska Native populations, and exacerbate health disparities. The 2020-2024 syphilis outbreak illustrates how restricted data access impedes effective public health responses. These practices represent a source of structuralized violence throughout the US public health system that contributes to the data genocide of American Indian and Alaska Native populations. Good governance practices like transparent data practices and the establishment of a social license (ie, the informal permission of a community to collect and use data) is essential to ethically balancing collective well-being with individual privacy in public health.

## Introduction

Advancements in digital technology have transformed the potential for real-time global data sharing, including in the field of public health. However, sharing data for public health use remains a challenge. Legal, relational, and technical barriers can impede access to the data that enable public health agencies to act for the benefit of their communities. [1-3] Most of these barriers are at least indirectly associated with a need to protect the privacy of data subjects.

Public health data custodians are acutely aware of these risks, but sometimes zealous overprotection of individuals’ privacy can harm communities [4]. In the case of American Indian and Alaska Native communities, a lack of access to public health data, data they are legally entitled to have, results in unnecessary deaths in these communities [5,6].

While this paper focuses on the ethical basis for sharing data with Tribes for public health activities, it is important to acknowledge the robust legal foundation for such action, including the treaties between the United States and various Indian Tribes that help round out the obligations of the federal government to Indian Tribes and Tribal people [7]. According to a long line of federal court cases, interpreting the Constitution, statutes, and treaties, the United States Congress has plenary and exclusive authority over Indian affairs. Within these cases, the Supreme Court has consistently held that “federal law preempts state law in the context of Indian affairs” [8]. Although Tribes and states coexist as distinct sovereigns, the legal doctrine of federal preemption requires that “Congressional power over Indian affairs supersedes conflicting state laws or state constitutional provisions” [8].

For example, when the Indian Health Care Improvement Act (IHCIA) and its subsequent amendments were enacted into law, this was construed as the federal government saying it was now preempting state action in the areas staked out within the IHCIA. Among other things, the IHCIA affirmed the trust responsibility of the federal government “to ensure the highest possible health status for Indians and urban Indians and to provide all resources necessary to effect that policy” [9,10]. Currently, the 12 Tribal Epidemiology Centers (TECs) provide epidemiologic and public health services to approximately 574 Tribes, 41 urban Indian organizations, and 9.7 million American Indian and Alaska Native people, and data are a necessary resource to support these activities [11]. The federal trust responsibility embedded in the IHCIA implicitly provides legal support for public health data sharing with Tribes and TECs as well as the ethical imperative to do so.

Federal public health law relating to Indian Tribes and federal services for Indian Tribes firmly supports Tribal governmental and TEC authority to collect and use data to promote public health [12]. This includes laws that empower governments to promote public health (eg, police powers), federal Indian law (eg, the Indian Self-Determination and Education Assistance Act of 1975 that established the federal policy of Tribal self-determination), and Tribal laws (eg, constitutions, codes, and case law) that support and shape Tribal public health authority [12].

As O’Connell and Abourezk [10] describe in detail, there is a strong federal legal foundation supporting the sharing of public health data with TECs due to their status as legally recognized public health authorities. Federal statutes that govern the receipt of identifiable data (ie, protected health information) by public health authorities from entities covered under the HIPAA (Health Insurance Portability and Accountability Act) (such as hospitals and medical clinics) apply to Tribes, TECs, and non-Tribal public health authorities.

Data access and use by Tribal governments and TECs is, therefore, legally distinct from the access and use of public health data by other entities that may work with or represent minoritized communities such as community nonprofit organizations. Tribes are sovereign nations that have the inherent right to govern their own resources, including data (ie, Tribal data sovereignty) in accordance with their cultural values and legal frameworks [13]. Restriction of data from Tribal governments, in particular, represents a failure to honor their legal status as sovereign entities that have been recognized since first contact with colonizers.

While federal and Tribal laws overwhelmingly support data sharing with Tribal public health authorities, Tribal governments, and TECs have long experienced difficulty in obtaining data from covered entities and their state and federal public health partners [10,14]. These data are central to effective public health practice and necessary for Tribal governments and TECs to fulfill their legal and ethical duties to promote health and eliminate disparities in American Indian and Alaska Native communities [15].

A 2022 US Government Accountability Office (GAO) report entitled, “Tribal Epidemiology Centers: HHS Actions Needed to Enhance Data Access,” documents the difficulties experienced by TEC personnel in accessing essential public health data [15]. The GAO report found that access to and use of data varies by TEC with many TECs reporting challenges related to accessing data from the Centers for Disease Control and Prevention (CDC), Indian Health Service (IHS), and states. The report found that the work that TECs can perform and the information they can in turn provide to relevant public health decision makers is determined by their access to needed data. Consequently, when data access is limited or inconsistent, so too is the ability to address the health needs of Tribal communities. The report found that the US Department of Health and Human Services (HHS) lacked policies, procedures, and guidance related to existing data-sharing systems and agreements that resulted in barriers to Tribal HHS data access. The GAO report provided 5 recommendations for enhancements to HHS and IHS policies, procedures, and guidance to improve TEC access to public health data [15]. While HHS has taken steps to address some of these recommendations, Tribal governments and TECs still face barriers to accessing data from their state public health partners.

The consequences of these long-standing impediments to data access were tragically highlighted during the COVID-19 pandemic [5]. The failure of state and federal health departments to share timely and complete public health data during the COVID-19 response was highlighted in the 2021 Urban Indian Health Institute report, “Data Genocide of American Indians and Alaska Natives in the COVID-19 Data” [5]. A total of 15 states reported no American Indian and Alaska Native cases on their COVID-19 dashboards, and many lacked complete racial information on the fraction of cases reported to the CDC, making accurate monitoring of the COVID-19 pandemic in American Indian and Alaska Native communities impossible [5].

However, the consequences of these impediments to full data access predate and extend far beyond the COVID-19 pandemic. The prolonged dearth of actionable public health data has hampered the ability of Tribes and TECs to combat other common and preventable public health challenges and have contributed to staggering disparities [6,16]. Tribal governments and TECs encounter systemic policy, bureaucratic, and relational barriers such as excessive privacy restrictions, data suppression practices, and paternalistic policies that impede their access to critically needed public health data.

In this paper we explore how these barriers perpetuate health inequities, examine the ethical dimensions of data sharing, and argue for a “share by default” model that aligns with public health ethics, respects Tribal sovereignty, and ensures the provision of timely, actionable information for American Indian and Alaska Native communities. Specifically, we assess the ethical and real-world implications of the status quo of data sharing impediments that exist between federal and state public health authorities and their Tribal public health and governmental partners. Currently, compared with their other public health authority counterparts, Tribal governments and TECs experience inequitable access to public health data, which severely impairs their ability to ensure the well-being of their constituent communities, fulfill their legally recognized role and responsibilities, and implement legal interventions and policy solutions that are informed by the best available public health data. While access to needed data is a challenge shared by nearly all public health authorities, the real, perceived, and sometimes intentional data-sharing barriers encountered by Tribal governments and TECs can be fairly described as structural violence perpetrated on Tribal communities.

To illustrate the deadly consequences of current data-sharing practices, we highlight the resurgence and prevalence of syphilis, a preventable and treatable disease, that has devastated American Indian and Alaska Native communities that do not have access to critically needed data that are readily available to their state and federal public health partners to effectively target preventative interventions and resources. These inequities in data access and data sharing represent an abdication of ethical responsibilities across the US public health system.

## An Ethical Imperative to Share Data for Public Health Activities

Public health ethicists have long argued that there is an ethical duty to share public health data where there is a demonstrated public health need [17-19]. This ethical duty is essential to fulfilling the central purpose of public health surveillance; quickly and efficiently supplying information to decision-makers for population health.

For example, the 2017 World Health Organization (WHO) ethical guidelines on public health surveillance state, “[w]ith appropriate safeguards and justification, those responsible for public health surveillance have an obligation to share data with other national and international public health agencies” [17]. Other organizations have similarly spoken on the imperative to share data. The International Association of National Public Health Institutes called for sharing “public health surveillance data by default” and “with as few restrictions as possible” [18]. Lee et al [20] extended this point, arguing that “not using [public health] data for improving health must be justified” [20]. Both the American Public Health Association (APHA) Code of Ethics and the Public Health Leadership Society Principles for the Ethical Practice of Public Health urge prompt and appropriate data dissemination to enable public health actions [21,22]. Pisani and AbouZahr [23] argue that sharing public health data collected with public resources is important to the maximization of the public benefit from those costs.

All this underscores that collecting data without dissemination to affected communities is itself unethical. The APHA Principles of the Ethical Practice of Public Health states, “Public health institutions should provide communities with the information they have that is needed for decisions on policies or programs and should obtain the consent of the community for implementation” [22].

Certainly, as Langat et al [19] argue, there are important reasons for limiting data sharing including considerations of (1) data property and ownership, (2) just distribution of benefits and burdens, and (3) the competitive ethos of contemporary science [19]; however, they also note that these reasons are each outweighed by ethical considerations that support data sharing.

Moreover, an ethical right to be counted can countervail a right to privacy in public health contexts [24]. Accordingly, the WHO Guidelines on Ethical Issues in Public Health Surveillance recognize that there is an affirmative obligation that governments “develop appropriate, feasible, sustainable public health surveillance systems” [17]. Public health data showing an existing harm or imminent danger can shine a light on these threats and empower individuals and communities to compel a governmental response. Therefore, individuals and communities, from Love Canal, NY to East Palestine, OH, often demand to be counted [24-26]. Counting (ie, public health surveillance) informs resource allocations, and inequitable counting results in inequitable resource distributions [27]. In the context of public health data sharing, this ethical right to be counted is frustrated when barriers (or refusals) to share adequate data interfere with the ability of a government to collect the data necessary to carry out its obligations to care for its constituents.

Existing data sharing barriers that impede Tribal access to these data, whether legal, relational, political, bureaucratic, or willful, are ethically dubious at best. Instead, public health ethics imposes a default obligation to share data with public health authorities with as few restrictions as possible when there is a public health need.

## Systemic Ethical Failures: Policies, Practices, and “Street-Level Bureaucrats”

Broadly, we argue that the US public health system is rife with deviations from these public health ethical imperatives [4,28,29]. These deviations manifest themselves in a variety of ways including in laws, policies, organizational practices, and discretionary decision-making that prohibits, unduly burdens, or otherwise limits ethical data sharing or access to needed data [4,30].

Here it is important to distinguish between law and ethics. Laws relate to what can or must be done, and ethics deals with what should be done. Importantly, what is ethical is not always legal, and what is legal is not always ethical [31]. The APHA Code of Ethics implores public health practitioners to examine and deliberate such ethical considerations in the policy-making process [21]. Public health practitioners are silently complicit to injustice when they do otherwise [21].

In some cases, laws or policies (particularly at the state level) clearly restrict the authority of a public health data custodian to share data [30]. For example, Montana Code § 50-16-603, which strictly restricts disclosures of health information controlled by the public health authorities within the state, permits disclosures of protected information for public health purposes but only lists other state or local public health authorities. The omission of Tribes and TECs from the exhaustive list of recipients in the text of the law constitutes a barrier to ethical data sharing with Tribes. In this instance, the deviation of law from public health ethical imperatives is relatively transparent. This means that the ethical obligation to address the unethical law or policy is also transparent to every person who cites the law as a barrier to ethical conduct [21].

Beyond these transparent structural barriers, deviations from ethical imperatives frequently derive from opaque organizational and individual decisions [32]. The intergovernmental data-sharing landscape exemplifies this issue, characterized by “striking legislative minimalism” [32]. Consequently, data-sharing decisions are often left to the discretion of “street-level bureaucrats,” with relatively low-level government agents wielding significant influence over agency actions [32]. The opacity of these decision-making processes arises from a variety of factors including data-sharing negotiations that may be shaped by (real or perceived) policies, [30] legal agreements that are not publicly accessible [33], decisions that may be hindered by unnecessarily burdensome processes that undermine public health data-sharing goals or that may be swayed by individual risk aversion, personal judgments, or interpersonal dynamics that are sometimes rooted in racism or paternalism. In each of these situations, the deviation from ethical imperatives is a product of the (often discretionary) actions of individuals and organizations within the US public health system.

Regardless of whether the ethical deficiencies are dictated by express policy provisions or products of individual or organizational actions, accepting the status quo is untenable. Such impediments and interference can amount to structural violence on native communities and are contrary to public health ethical imperatives [1,17,19,20,34,35]. Unethical policies provide only a false aegis to culpability for the individuals or organizations that cite them as justification for insufficient data sharing. Public health ethical imperatives demand actions to revise existing policies and practices that impede the sharing of timely and adequate data to Tribal public health organizations.

## Outdated Approaches to Privacy Risks Impede Community Health Interests and Public Health Objectives

Privacy considerations are oft cited as a reason to withhold or redact public health data. These privacy considerations expressly or implicitly rest on bioethical concerns that have long been rejected in public health applications [1,17,35,36]. For example, consent has been the primary tool used to support and protect privacy interests in research and clinical contexts [37]. But, in public health contexts, overzealous protection of individual privacy harms the community. This is one reason why the HIPAA Privacy Rule includes a generous public health exception permitting public health use of protected health information without individual consent.

## Misguided “Protections” That Cause Greater Harm

Tribal government and TEC efforts to access needed public health data are often frustrated by the misguided efforts of state and federal public health authorities to mitigate privacy risks from data sharing including discretionary deidentification, data suppression, and unnecessarily burdensome administrative access procedures.

## Deidentification Can Contribute to Harm in Public Health Contexts

Deidentification can be a problematic approach to mitigating privacy risks in many public health contexts because it can cause both individual and group harm that offsets its benefits [38-40]. In the absence of informed consent, data controllers frequently rely on deidentification before sharing data.

There are 2 principal motivations for deidentifying data [27]. First, laws might allow data to be used or shared with fewer restrictions if deidentified. Second, deidentification is often used as an ethical precaution (as opposed to a legal requirement) to reduce the potential risks to data subjects (eg, when HIPAA covered entities insist on deidentifying data before disclosing protected health information pursuant to the research exception that does not legally require deidentification) [38]. But, when not legally required, the risks of deidentification must be weighed against the anticipated benefits [27]. This is why public health ethical guidelines expressly support the collection of identifiable data even in the absence of express consent under certain circumstances.

For example, the 2017 WHO [17] ethical guidelines state, “In some instances, the collection of names or identiﬁable data is both technically and ethically imperative.” The guidelines expressly mention outbreak investigation, case follow-up, and preventive response activities as instances where disclosing names and other identifying information is appropriate [17]. Deidentification directly frustrates these important public health objectives by removing essential information, reducing the usefulness of information to provide the basis for action [41]. Moreover, the deidentification process consumes precious time so that when deidentified information is eventually shared its value for situational awareness is also diminished. Together, WHO Guidelines 11 and 14 (ie, the obligation to share data with other public health agencies, discussed above) state that there is a firm ethical duty for federal, state, and local public health agencies to share identifiable public health data with their Tribal counterparts when there is a public health need [17].

In fact, individual protections, like deidentification, often only shift the risks posed by data usage, rather than mitigate them [38,42]. For example, federal, state, and local health department practices that routinely deidentify public health data before sharing those data with Tribes or TECs for public health purposes will almost always suppress data about uncommon characteristics to reduce the risk of identifying individuals with those characteristics. This practice disproportionately affects Tribal communities. For instance, a public health agency that suppresses “small cell sizes” (a common deidentification practice) before sharing public health data will disproportionately suppress data on Tribal members because of the relatively small population of American Indian and Alaska Native people in any given area. In this way, deidentification practices contribute to social structures that systematically obscure the accurate assessment of health disparities in Tribal communities, ultimately leading to undercounting and underallocation of critically needed resources and support.

The assumption that deidentification reduces risks by rendering a data subject more difficult to identify is increasingly questionable in big data contexts [43]. For example, often the objective in big data applications is to gain group insights that can be both helpful or harmful to groups and the individuals that comprise them [43]. Deidentification cannot, however, eliminate all group risks but often will aggregate them. Thus, deidentification may result in real harm with little community health benefit.

Deidentification can also cause individual harm. Identifiable data are vital to helping public health agencies accurately monitor the spread of disease, identify persons at risk (eg, contact tracing), and target individual interventions [40]. When data holders deidentify the data provided to Tribal public health authorities, Tribes and TECs are unable to perform these basic public health interventions. The deidentification practices that are intended to protect individuals can impede the benefits that individuals can derive from these public health activities.

## Data Suppression Hobbles American Indian and Alaska Native Epidemiology

Some US public health authorities that share data with Tribal public health partners exclude data on nonnative individuals, a precaution that is dangerous and problematic.

This practice directly contradicts public health ethical guidance in several ways. First, excluding nonnative persons from public health data sharing is bad epidemiological science that contradicts public health ethical guidance to share public health data. Excluding nonnative individuals from a dataset biases the data and denies Tribal epidemiologists comparison groups and the critical context needed to interpret the statistics. Informed public health decision-making requires this missing context. Moreover, excluding nonnative individuals from shared data effectively makes the identification and quantification of disparities impossible.

Second, excluding nonnative individuals from data shared with Tribal public health partners hides the health impacts that result from the interactions between native and nonnative individuals. In a world where disease regularly ignores geographic, political, and cultural boundaries, this type of data suppression simply makes no public health sense and amounts to a substantial barrier to understanding present and emerging public health threats. Excluding nonnative data makes even less sense given that many nonnative persons live on Tribal lands. Thus, excluding nonnative data from data shared with Tribal public health partners has harmful consequences for everyone.

Third, identifying Tribal members within state or local epidemiological datasets for the purpose of excluding all nonnative persons from data sharing needlessly complicates and delays data sharing. Existing electronic health records rarely record Tribal membership due to inadequate data standards, (ie, an absence of structured data fields for Tribal affiliation and inadequate documentation practices). Consequently, health departments that attempt to filter all nonnative persons from disclosures to Tribal organizations embark on a complex and protracted process that will delay timely data sharing. While we acknowledge that there is a tremendous benefit to developing data standards and systems that enable better Tribal affiliation documentation and demographic categorization, such actions are not prerequisites to better data-sharing practices.

Therefore, where federal, state, or local public health agencies refuse to share the nonnative data needed for good epidemiological investigations, they are deviating from public health ethical guidance to freely and promptly share public health data [1,17,19,20,34,35].

## Administrative “Protections” That Amount to Data Discrimination

In contrast with the view of ethicists that public health data should be shared “by default” and “with as few restrictions as possible,” [18] Tribal governments and TECs encounter administrative hurdles when they attempt to access public health data [15]. For example, the GAO report found that IHS required TECs to submit “Freedom of Information Act” requests, the same time-consuming process required of the general public, to access needed public health data [15]. This problem is not isolated to the federal government, as 3 TECs reported that restrictive state rules, among other reasons, prevented their access to any state data [15].

Unequal access to public health data for Tribal compared to non-Tribal public health authorities is a discriminatory practice. Yet, that is the status quo in the United States, despite the existence of equal federal legal authority to access data and perform public health functions [10].

As constructed, the current public health data sharing system de facto excludes Tribes from using public health data and results in Tribes having to seek these data from non-Tribal public health entities [5,15]. This system is likely a result of the historical paternalist and colonialist attitudes of the United States and state governments that consider Indian Tribes and their members incapable of being actively involved in their own governance. These stereotypes and inaccurate assumptions about Indian Tribes have prevented full Tribal participation in a range of governmental activities including in public health.

Unfortunately, these attitudes persist today and can impede non-Tribal public health authorities from providing life-saving data to Tribes when requested. So, Tribes and TECs face a double bind as they are excluded from the public health data collection system used by non-Tribal public health authorities and attempts to access the public health data system are blocked by the refusal of non-Tribal public health authorities to share data.

While some might argue these administrative burdens might be necessary to protect individual privacy interests, this too is a false aegis. In truth, these discriminatory administrative policies and processes result in persistent structural violence that contributes to systemic health disparities and deaths in American Indian and Alaska Native communities with little real benefit to individuals.

## Case Study: Syphilis Epidemic (2020-Current)

The syphilis crisis in American Indian and Alaska Native communities (2020-2024) is an illuminating example of the failure of current data-sharing practices. Syphilis is a completely preventable disease that has been curable for nearly a century, and yet cases have risen dramatically and disproportionately in American Indian and Alaska Native populations as compared to other populations and demographic groups. Inadequate sharing of health data with Tribes, in stark contrast to ethical imperatives, is a significant barrier to Tribal government and TEC efforts to address this crisis [44].

**Figure 1:**
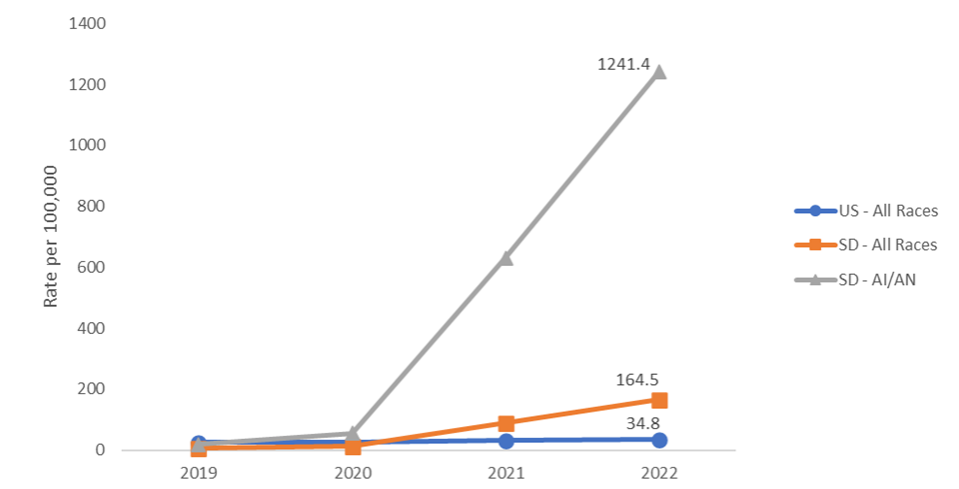
Rates for Early Syphilis (per 100,000), US, South Dakota, and South Dakota American Indian and Alaska Native (2019-2022).

For instance, South Dakota has seen a drastic increase in syphilis and congenital syphilis cases since 2020 (Figure 1 and Multimedia Appendix 1) [45,46]. To put these data in context, an 80% rise in national rates of syphilis between 2018 and 2022 justified the creation of a national task force For comparison, between 2019 and 2022, syphilis rates among American Indian and Alaska Native populations in SD surged by 6821%, reaching over 35 times the 2022 national rate. Yet, both the state department of health and federal IHS have been reluctant to provide protected health information to affected Tribes or the TEC that serves them. Because these agencies refuse to provide Tribes with needed public health data, Tribes have been unable to fully assist in the response.

At the direction of the 18 Tribal leaders of the region in March 2023, the Great Plains Tribal Epidemiology Center (GPTEC) requested a CDC Epidemiological Aid (Epi-Aid) to assist with the syphilis outbreak. Due to the challenges that GPTEC encountered in accessing data on syphilis cases, a key objective of the Epi-Aid was to “[c]haracterize syphilis cases among Tribal populations in the [GPTEC] catchment area” (CDC, report to GPTEC, January 22, 2024). Despite CDC involvement and advocacy, the data required to complete the Epi-Aid was not provided by all relevant state health departments until November 2023, months after the CDC team deployment. While the deidentified data needed to conduct the report was ultimately provided, the delay in accessing the data contributed to the final report being published in January 2024, 6 months after the deployment. This rapid response action offered by the CDC took nearly a year to complete due to the data access challenges encountered by GPTEC. In that time syphilis continued to spread with 46 infants being diagnosed with congenital syphilis in South Dakota between March 2023 and January 2024, of whom 41 (89%) were American Indian and Alaska Native [45]. While syphilis is certainly concerning in the adult population, it can be deadly in newborns. The tragedy of this outbreak could have been mitigated if data-sharing practices were aligned with public health ethics.

Data are often shared with Tribes only after being deidentified. However, contact tracing, which is critical to managing outbreaks, is impossible without knowing who is infected. As a result, the deidentification standards that were intended to protect individuals effectively denied them essential preventative and treatment services.

In April 2024, the CDC was again deployed to the region at the request of GPTEC to assist in contact tracing. Due to a data sharing agreement signed with the state of South Dakota in March 2024, GPTEC and South Dakota Tribes had access to identifiable data on people who had been diagnosed with syphilis or may have syphilis. In only 8 days of fieldwork teams of Tribal, TEC, CDC, and IHS staff members in 4 areas of the state were able to treat 62 people for syphilis, including 6 pregnant people (CDC, letter to GPTEC, May 28, 2024). The most significant difference between these 2 deployments was access to identifiable data.

When Tribal health authorities are unable to effectively respond to preventable infectious disease outbreaks, all people are put at greater risk. Though the syphilis outbreak spread nationally, the highest rates continue to be in South Dakota. If GPTEC and Tribes had access to data before the start of the outbreak consistent with ethical guidelines, they would have been able to better address it with contact tracing and other public health measures. The delay in data access—and continued lack of access in much of the great plains area—contributed to the high rates of syphilis in the region and, therefore, the country. Ethical data access for Tribes and TECs protects the health of all Americans.

## A Better Way to Protect: Good Governance and Social License

In public health contexts, it is appropriate to take a group-based approach to the “respect for persons” ethical principles (as opposed to the individual-based approach of bioethics) [17]. For example, Megan Doerr argues that building or establishing a “social license” for collecting and using data can be more appropriate and protective than deidentification [47]. A social license represents the informal permission of a community to engage in a specific activity [48,49]. In public health contexts, a social license can legitimize the collection, use, or sharing of data about relevant communities. Similarly, WHO ethical guidelines for public health surveillance highlight the importance of involving affected communities in the decision-making process [17]. Developing a social license is essential to ensure that the trade-off between individual and collective interests is appropriate and ethically sound [43].

State and federal public health agencies established their social license to conduct public health surveillance through democratic processes, robust government accountability measures, and an understanding that the data collected will be used only to protect community health. Similarly, Tribal governments and TECs have a social license to collect and use public health data about their Tribal members established through their own sovereign governance and accountability mechanisms.

In fact, Tribes have substantial experience in the practices and activities that support social license for data collection and use. Tribal data sovereignty and indigenous data governance have emerged as widely recognized movements for Indigenous rights, self-determination, and ethical data management across academic, governmental, and community-based settings [13]. These movements were impelled, in part, by the unique risks resulting from the use of native data by nonnative persons or organizations that might not appropriately consider the harms or stigma that might result [50]. Yet, these movements are grounded in fundamental principles and rights inherent in sovereignty and personhood that likewise give power to social licenses as protective mechanisms for data collection and use. In particular, indigenous data governance practices and frameworks (eg, Collective benefit, authority to control, responsibility, and ethics Principles for Indigenous Data Governance) present compelling examples of how to effectively build and support social license in practice through relational accountability, stewardship, and respect for community values [13,51].

However, data on American Indian and Alaska Native individuals and Tribal members are often spread across jurisdictional lines, making data collection for Tribal organizations and TECs inherently more complex legally and practically than is the case for federal or state public health organizations. Obtaining the data necessary to fulfill their social license to use the data to promote population health requires the cooperation of federal and state public health partners.

Good governance and data stewardship are essential to support and nurture social license [52]. Public health authorities are effectively population fiduciaries who make decisions and take actions based on the best interests of the populations that they serve [53]. As such, privacy practices that prioritize the rights of individuals cannot be the only focus of public health agencies. Confidentiality protections provide an alternative approach to data governance that can permit public health authorities to use data more freely as population fiduciaries while still restricting inappropriate data uses. Similarly, adequate security measures can reduce risks of inappropriate access to or use of data while simultaneously permitting appropriate data uses. By shifting data protection governance strategies toward a population fiduciary strategy encompassing robust confidentiality and security practices, public health authorities can better align their data governance with public health ethics.

Nurturing a social license requires that data collection, its uses, and its governance are subject to public scrutiny that promotes data holder transparency and accountability that includes data dissemination to affected communities [17]. Above all, good governance must be grounded in ethical principles that do not ignore obligations to share public health data.

## Discussion

The guidance of public ethicists in making public health data readily accessible between public health partners where there is a public health need provides a stark contrast to the status quo. Often Tribes—sovereign entities with primary public health responsibility for their constituent communities—are not consulted on state or federal public health data collection from Tribal communities and are not provided with the collected data needed to make crucial decisions and take needed action. This lack of consultation and provision of collected data results in 3 significant consequences. First, it wrongly subordinates Tribes to state and federal public health partners. Second, it restricts the ability of Tribal public health authorities to promote public health and limits Tribal self-determination. And third, it results in the misallocation of resources, leaving Tribes guessing to the nature and magnitude of public health issues and unable to make data-informed decisions. Data collection wastes time and physical resources when it is not accompanied by the full dissemination critically needed by affected communities. For these reasons, the current default of not sharing data between federal, state, and local public health authorities and their Tribal partners cannot be ethically justified.

In some ways, federal Indian law interacts in unconscionable ways with the state of inadequate public health data availability experienced by Tribes. For instance, some governmental officials may conclude that the federal policy of Tribal self-determination absolves federal, state, and local governments of responsibility for threats to American Indian and Alaska Native public health. Yet, depriving Tribes of the public health data they need to protect themselves only ensures their continued vulnerability to increasingly existential threats. In this way, the status quo of inadequate data sharing with Tribes is just one dimension of a complex system of continued structuralized violence that contributes to the ongoing data genocide of American Indian and Alaska Native peoples [54].

There are meaningful steps that US public health agencies can take to address these systemic failures. Public health agencies at all governmental levels should:

1. Critically examine their data-sharing laws, policies, and practices to ensure that they are consistent with the ethical mandates of public health surveillance and action.

2. Bring any legal impediments to ethical data practices to the attention of policy makers (e.g., the absence of express language in laws stating that Tribal governments and TECs are permitted recipients of identifiable public health data). At a minimum, data-sharing practices with Tribes and TECs should be equivalent to other public health agencies [54].

3. Revise policies to align with a “share by default” approach, consistent with public health ethical guidelines, including removing data pre-processing practices (eg, de-identification, small-cell suppression, removal of non-native records) that delay the sharing of timely public health data and reduce the usefulness of the data that is shared. Consistent with Lee, Heilig, and White, decisions not to share timely data with Tribes should be justified, and justifications should expressly address the anticipated harms of not sharing data [20].

4. Adopt standard mechanisms for Tribes and TECs to request identifiable public health data with transparent and pre-defined terms for data transmission, use, and governance, consistent with public health ethics.

5. Ensure that public health data collection, sharing, and use practices respect tribal sovereignty. As Tribes are sovereign nations, their requests for data should be treated differently than requests from non-governmental groups. Public health agencies should create processes that honor Tribes as sovereign entities.

6. Establish and maintain regular communication with Tribes and TECs. Adopting policies and practices consistent with the federal guidelines on Tribal consultation can help public health agencies better understand both Tribal data needs and the barriers they encounter in achieving adequate data sharing [54].

## Conclusion

Without aggressive actions to reverse the status quo, the US public health system will continue to be complicit in the data genocide of American Indian and Alaska Native communities. Public health authorities must adopt policies that expressly require data sharing with Tribes and TECs to limit the impact of discrimination and racial bias that leads to inequitable data access.

## Appendix

Multimedia Appendix 1 Rates for Early Syphilis (per 100,000), U.S., South Dakota, and South Dakota AI/AN (2019-2022).

## Abbreviations

APHA: American Public Health Association
CDC: Centers for Disease Control and Prevention
Epi-Aid: CDC Epidemiological Aid
GAO: Government Accountability Office
GPTEC: Great Plains Tribal Epidemiology Center
HHS: Department of Health and Human Services
HIPAA: Health Insurance Portability and Accountability Act
IHCIA: Indian Health Care Improvement Act
IHS: Indian Health Service
TEC: Tribal Epidemiology Center
WHO: World Health Organization

## Data Availability

All data generated or analyzed during this project are included in the published article and Multimedia Appendix 1.
