# Supplementary material for: Dying in Darkness: Deviations From Data Sharing Ethics in the US Public Health System and the Data Genocide of American Indian and Alaska Native Communities
Source: J Med Internet Res. 2025 Mar 26;27:e70983. doi: 10.2196/70983 (PMC11982748; doi:10.2196/70983)
Supplement: Multimedia Appendix 1 [file jmir_v27i1e70983_app1.docx]

Table S1: Rates for Early Syphilis (per 100,000), U.S., South Dakota, and South Dakota AI/AN (2019-2022).

| Early Syphilis Rates | 2019 | 2020 | 2021 | 2022 |
| --- | --- | --- | --- | --- |
| US - All Races | **24.6** | **25.6** | **31.8** | **34.8** |
| SD - All Races | 6.3 | 11.7 | 88.1 | 164.5 |
| SD - AI/AN | 18.2 | 55.6 | 630.3 | 1241.4 |

Case count data sourced from:

1. Infectious Disease Surveillance. South Dakota Department of Health. 2024. <https://doh.sd.gov/health-data-reports/infectious-communicative-disease-data-reports/infectious-disease-surveillance/> (accessed 25 November 2024)
2. Centers for Disease Control and Prevention. Table 12. Total Syphilis – Reported Cases and Rates of Reported Cases by State/Territory and Region in Alphabetical Order, United States, 2017-2021. Cdc.gov. 2023.
